# Cost benefit analysis for green hydrogen production from treated effluent: The case study of Oman

**DOI:** 10.3389/fbioe.2022.1046556

**Published:** 2022-11-25

**Authors:** Hind Barghash, Arwa Al Farsi, Kenneth E. Okedu, Buthaina Mahfoud Al-Wahaibi

**Affiliations:** ^1^ Department of Engineering, German University of Technology, Muscat, Oman; ^2^ Department of Electrical and Communication Engineering, National University of Science and Technology, Muscat, Oman; ^3^ Department of Electrical and Electronic Engineering, Nisantasi University, Istanbul, Turkey; ^4^ Research and Development, Oman Water and Wastewater Service Company (OWWSC), Muscat, Oman

**Keywords:** cost benefit analysis, electrolysis, green hydrogen, treated effluent, wastewaters

## Abstract

Recently, the management of water and wastewater is gaining attention worldwide as a way of conserving the natural resources on the planet. The traditional wastewater treatment in Oman is such that the treated effluent produced is only reused for unfeasible purposes such as landscape irrigation, cooling, or disposed of in the sea. Introducing more progressive reuse applications can result in achieving a circular economy by considering treated effluent as a source of producing new products. Accordingly, wastewater treatment plants can provide feedstock for green hydrogen production processes. The involvement of the wastewater industry in the green pathway of production scores major points in achieving decarbonization. In this paper, the technical and economic feasibility of green hydrogen production in Oman was carried out using a new technique that would help explore the benefits of the treated effluent from wastewater treatment in Oman. The feasibility study was conducted using the Al Ansab sewage treatment plant in the governate of Muscat in Wilayat (region), Bousher. The results have shown that the revenue from Al Ansab STP in a conventional case is 7.02 million OMR/year, while sustainable alternatives to produce hydrogen from the Proton Exchange Membrane (PEM) electrolyzer system for two cases with capacities of 1,500 kg H_2_/day and 50,000 kg H_2_/day, would produce revenue of 8.30 million OMR/year and 49.73 million OMR/year, respectively.

## 1 Introduction

Humans have benefited from natural resources like the Sun, water, minerals, and vegetation over the years, by living and building their communities. Energy and water resources were mostly used to develop civilizations and industrial revolutions. However, in recent years the world noticed that many issues have occurred with these natural resources. Freshwater resources are being polluted and depleted, non-renewable energy resources are being consumed in enormous amounts, and many negative impacts have happened due to the excessive use of these resources that have contributed to global warming and climate change. Therefore, actions must be taken to change the world’s focus to a greener and more sustainable future. 

Since 1988, water resources in Oman are considered a national wealth that needs to be protected and conserved (Royal Decree No.82/88, n. d.). Water and wastewater management has become one of the main aspects to conserve the natural resources of Oman. There is a huge reliance on groundwater, as a natural resource, and desalination to provide potable and non-potable water in Oman due to its location in an arid region, with a lack of precipitation and freshwater resources. One of the drivers to achieve Oman’s 2040 vision is the introduction of water reclamation opportunities for sustainable growth, considering economic, social, and governance performance. This is part of the United Nations Sustainable Development Goals (UNSDGs) target to ensure the availability and management of water and sanitation. Hence, Oman was the first of the Gulf Cooperation Council (GCC) countries to establish regulations regarding wastewater through the Royal Decree 48/84 which included articles about the management of wastewater and sludge (Jaffar et al., 2017). Moreover, as Oman is located in an arid region with a lack of precipitation and freshwater resources, the country relies on groundwater as a natural resource and desalination to provide potable and non-potable water. The used water is transferred to wastewater treatment plants to remove contamination in order to help produce clean treated water.

Although Oman has an existing system of wastewater treatment, the full potential of the treated effluent of the wastewater treatment is yet to be tapped. The current practice is that the treated effluent produced is only reused for unfeasible purposes such as landscape irrigation, cooling, or it is disposed of in the sea. There are other reuse purposes that could be more beneficial by introducing a more feasible water reclamation technology. This will result in recovering large amounts of high-quality reusable water that can be used in many applications with the hope of generating high revenue and shorter payback periods in industrial applications, groundwater recharge, potable water, and energy generation. As a result, it will contribute to a new resource in fulfilling the demand for water, which is already threatened by the issue of water scarcity from the recovered clean water, and revenue could be generated as well.

One of the drivers to achieve and contribute to Oman’s 2040 vision is by introducing water reclamation opportunities for sustainable growth in three main pillars: economic, social, and governance (ESG) performance. The vision targets the UNSDGs as it aims to achieve them, with a focus mainly on goal six: ensuring the availability and management of water and sanitation for all (Oman vision 2040, n. d.). Furthermore, there are many drivers and calls for resource recovery in addition to the urge to implement a circular economy. In order to achieve the SDGs, environmental issues like ecosystem losses, seawater intrusion into groundwater, and temporary water shortages can be solved by water reclamation. This process would open doors for energy recovery as well, and mitigate the pressure of reducing greenhouse gas emissions in the wastewater sector (Kehrein et al., 2020; Barghash et al., 2021; Okedu et al., 2022).

In the literature, some work on the cost-benefit analysis was carried out for hydrogen production by comparing eight different technologies using the classical analytic hierarchy process (AHP) and the Fuzzy AHP (Sonal et al., 2014). The technologies employed in this study were steam methane reforming, coal gasification, partial oxidation of hydrocarbons, biomass gasification, photovoltaic-based electrolysis, wind-based electrolysis, hydro-based electrolysis, and water splitting by chemical looping. The study was evaluated using five criteria of greenhouse gas emissions, raw material and utility consumption, energy efficiency, scalability, waste disposal, and atmospheric emissions. The study concluded that fossil fuels have fewer benefits with many environmental impacts, though they were more cost-effective. In another study carried out by Hui et al. (2021), on the urgent need for selecting sustainable hydrogen production technologies (HPT), the life cycle sustainability was evaluated from economic, environmental, social, emergy-based, and technical dimensions. The results of the study show hydrogen production from copper-chlorine (Cu–Cl) thermochemical water-splitting is the most sustainable technology option, followed by water electrolysis *via* wind power and natural gas steam reforming. The use of coal gasification is the least sustainable option. In another study, by Barghash H. et al. (2022), a life cycle assessment study for bio-hydrogen gas production from sewage treatment plants using solar PVs was carried out. In this study, hydrogen gas was produced by 1L of sludge and treated effluent (TE) by several methods using a reactor, with a volume of 0.96 H_2_ L/L media. Also, the Life Cycle Impact Assessment (LCIA) process was used to study resource depletion, the ecosystem, and human impacts, and efforts were made to reduce the negative impacts by implementing several solutions. The OpenLCA software was used as a tool for computing the impacts along with the Eco-invent database. A comparison of the LCIA with and without the use of solar energy was carried out also in the study. The results reflected that the use of hydrogen gas with a solar energy system would help achieve optimal solutions in mitigating carbon footprints.

There are various ways of producing hydrogen in the literature, such as biological processes that extract energy from biomass (Kazimierowicz, et al., 2022; Khorasani, R., et al., 2021; Dudek et al., 2022; IRENA, 2020). The idea of electrically splitting water molecules is 260 years old, however, the principle of using electrolysis to produce green energy arose in the 1990s. This idea was adopted due to the increased concern about tackling environmental issues (Smolinka et al., 2022). This current study introduces a new idea to decarbonize one of the important sectors in society which is water and wastewater services and reduce the industrial reliance on wastewater to produce energy. This can be achieved by utilizing the treated effluent into producing green hydrogen. The salient part of this paper is hydrogen production from treated effluent and sewage sludge that were employed as reuse applications. Hydrogen is one of the eco-friendly energy sources that has rapidly gained attention in recent years because it relies on water to be produced. It can be characterized through different color spectrums depending on its source. The most sustainable hydrogen is green hydrogen which is produced from renewable energy and has no greenhouse gas emissions. In this paper, the technical and economic feasibility of green hydrogen production in Oman was carried out. An overview of wastewater treatment and green hydrogen production was discussed. The feasibility study was conducted using the Al Ansab Sewage Treatment Plant (STP) which is managed by Oman Water and Wastewater Services Company (OWWSC), located in the governate of Muscat in Wilayat (region) Bousher. The study was evaluated using sustainable alternatives to produce hydrogen from a Proton Exchange Membrane (PEM) electrolyzer system for two cases with capacities of 1,500 kg H_2_/day and 50,000 kg H_2_/day. This study could help in achieving a circular economy by considering treated effluent as a source of producing new products. Furthermore, the study could help stakeholders and policymakers in wastewater industries forge a green pathway to achieving decarbonization.

## 2 Overview of wastewater treatment

Wastewater treatment is the process of removing and eliminating waterborne liquids and solids from the discharged used water to produce clean water called treated effluent that can be reused or recycled (Salgot & Folch, 2018a). Sludge is also produced as a by-product that is concentrated with the pollutants removed throughout wastewater treatment processes. Wastewater is contaminated water with different kinds of pollutants that have physical characteristics like strong odor, dark color, and high turbidity, chemical characteristics including high levels of chemical oxygen demand (COD), total organic carbon (TOC), nitrogen N, phosphorus P., etc, and biological characteristics such as biological oxygen demand (BOD) and microbial organisms. These characteristics differ depending on the source of wastewater, whether it is domestic, industrial, commercial, agricultural, or collected stormwater. The design of sewage treatment plants is planned to depend on the characteristics of wastewater and treated effluent.

**FIGURE 1:**
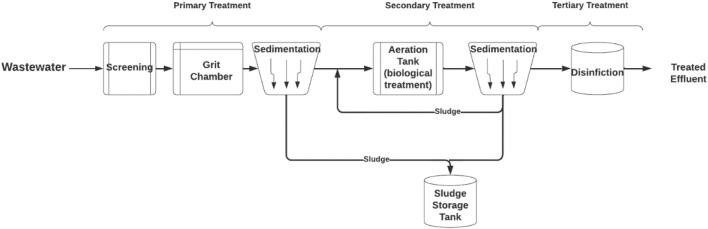
Wastewater treatment process.

The wastewater treatment stages are determined with specified processes of contamination removal as shown in Figure 1. Wastewater enters the primary treatment to remove inorganic larger-sized physical contaminants through grit removal and screening. Oils and grease are removed in the first sedimentation tank (Sonune & Ghate, 2004). Then, the water enters the aeration tank where a secondary or biochemical treatment process occurs. It requires more energy to maintain the interactions between microorganisms present in the effluent and nutrients in a controlled environment that allows the bacteria to feed on biological contaminants like food waste and feces. This is done through the use of activated sludge in the oxidation of dissolved organic matter which is then filtered from the process when the water enters the second sedimentation tank (Salgot & Folch, 2018b). The sludge enters a storage tank where it can be dewatered and reused for purposes such as creating compost.

Despite the water passing through primary and secondary treatments, it does not achieve the quality of water that can be reused for domestic, agricultural, or industrial purposes. Consequently, advanced treatment steps are additionally applied as a tertiary treatment process. It differs depending on the quality of the desired treated effluent and reuse purposes. For example, desalination technologies can be utilized to produce demineralized water such as reverse osmosis (RO), electrodialysis (ED), and ion exchange (IX) (Sonune & Ghate, 2004). There are two purposes for treating wastewater and constructing wastewater treatment plants. First, it is necessary for conserving the sanitation of cities by preventing the discharge of contaminated water into the environment. Releasing such contaminants into the environment can affect freshwater resources and wildlife, which could result in severe effects on human health. Secondly, treated effluent produced can be considered a reclaimed water source and reused in different applications (Salgot & Folch, 2018b).

## 3 Overview of green hydrogen production

Wastewater treatment plants are rarely considered a producer of new sources of energy, despite the fact that it produces treated effluent as viable water resources and has a rich microorganisms supply from the feed wastewater and the generated sludge. These factors can be merged into producing one of the most attractive sources of clean energy, hydrogen.

Hydrogen gas has gained a noticeable interest in recent years due to its ability to be produced from green sources and to work as an energy carrier. It can be an alternative energy source to fossil fuels as it is an eco-friendly source that does not emit any greenhouse gas elements (carbon, nitrogen, and sulfur) during the combustion process. It can be utilized in fuel cells to generate heat and electricity (Lin et al., 2012). Also, molecular hydrogen contains the highest calorific value among the known gaseous fuels per unit mass of 143 GJ/ton, while natural gas is only 44.2 GJ/ton (Show et al., 2011; UN STAT, 2014).

There are many methods to produce hydrogen. They differ depending on the source of energy. Thermochemical methods release hydrogen from organic fuels like natural gas and coal through heating and chemical reactions. Electrochemical methods like electrolysis use electricity to split the water molecule (H_2_O) into hydrogen and oxygen. Lastly, biological methods rely on microorganisms’ biological processes of photolysis and fermentation to produce biohydrogen (Hu, 2021).

## 4 Green hydrogen production using the electrolysis method

The treatment of wastewater has the advantage of being able to produce treated effluent with any desired quality. This can result in using treated effluent as a renewable water source for electrolysis in a unit operation called an electrolyzer. Eq. 1 shows that 1 mol of water requires 237.2 kJ of electricity to produce hydrogen and oxygen gases alongside heat of 48.6 kJ/mol.

$$H_{2}O+Electricity\;\left(237.2\frac{kJ}{mol}\right)\to Heat\;\left(48.6\frac{kJ}{mol}\right)+H2+\frac{1}{2}\;O_{2}$$ (1)

The electrolyzer’s technical characteristics involve three levels. The first level is the cell which includes two electrodes (anode and cathode) submerged in a liquid electrolyzer or adjacent to a solid electrolyte membrane, two porous transport layers, and bipolar plates to assist in inducing mechanical flow. The second level is the stack comprised of multiple cells connected in series. The third level is the scaled-up advanced system level, i.e. the design of the hydrogen production plant that includes operation equipment for cooling, hydrogen processing, electricity input source, water input source and treatment, and gas output (IRENA, 2020).

There are diverse types of electrolysis processes that differ depending on the electrolyte type, operating conditions, and ionic agents (O^−2^, H^+,^ and OH^−)^. The four types of electrolyzers are alkaline water electrolyzer (AWE), solid oxide electrolyzer (SOE), proton exchange electrolyzer (PEM), and anion exchange electrolyzer (AEM). Table 1 indicates the difference between the four types according to operating temperature, operating pressure, type of electrolyte, and separator. AWE and PEM are already used at the commercial scale while AEM and SOE are still being studied at the lab scale. AWE differs from the other technologies as it contains liquid electrolytes, while PEM, AEM, and solid oxide are filled with an electron-insulating solid electrolyte to transport the ions between the electrodes (Shiva Kumar & Himabindu, 2019; IRENA, 2020).

**TABLE 1:** Types of Electrolyzers and operating conditions (IRENA, 2020).

Operating conditions	AWE	PEM	AEM	Solid oxide
Operating Temperature	70–90°C	50–80°C	40–60°C	700–850°C
Operating Pressure	1–30 bar	<70 bar	<35 bar	1 bar
Type of electrolyte	5–7 mol/L potassium hydroxide (KOH)	PFSA membranes	DVB polymer support with KOH or NaHCO_3_ 1 mol/L	Yttria-stablized Zirconica (YSZ)
Separator	ZrO_2_ stabilized with PPS mesh	Solid electrolyte (PFSA membrane)	Solid electrolyte (DVB polymer with KOH or NaHCO_3_)	Solid electrolyte
				(YSZ)

**FIGURE 2:**
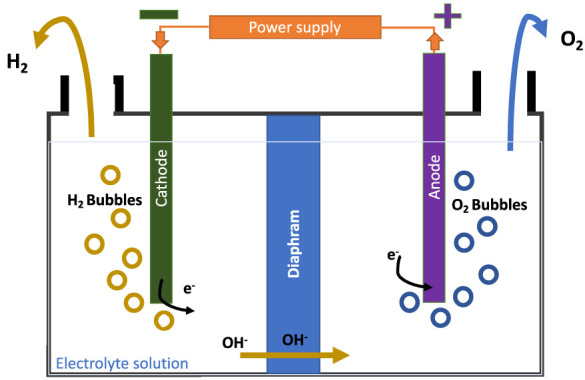
Alkaline electrolysis process.

Alkaline electrolysis is the basic type of electrolysis and the first one that was established commercially. It consists of two electrodes (anode and cathode) placed in an alkaline electrolyte, either KOH or NaOH as presented in Figure 2, operating at lower temperatures of 50–80°C. The membrane is a porous inorganic diaphragm placed in the middle of the cell to separate the anode and cathode. Also, this layer is used to transport the OH^`-^ ions to the anode side. Eqs 2, 3 represent the chemistry that occurs in the cathode and anode sides where the hydrogen gas is released from the cathode side. It has a simple system design and is easily manufactured at a large scale. However, the drawback of this process is that the hydrogen and oxygen gases produced have the possibility of mixing when passing through the electrolyte solution (Shiva Kumar & Himabindu, 2019; IRENA, 2020).

$$Anode:4OH^{-}\to 2H_{2}O+O_{2}+4e^{-}$$ (2)

$$Cathode\;:4H_{2}O+2e^{-}\to 2H_{2}+2OH-$$ (3)

**FIGURE 3:**
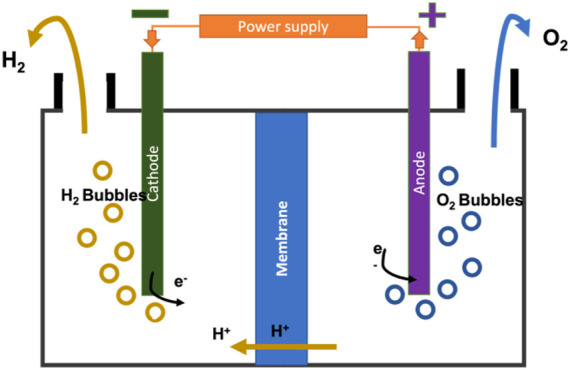
PEM electrolysis process.

Figure 3 illustrates a schematic view of one of the processes, PEM. It is designed to resolve the issue of the gas mixing in the electrolyte solution by replacing it with a solid electrolyte that requires higher pressure values than an alkaline electrolyzer. The PEM electrolyzer consists of an anode section and a cathode section separated by a membrane where the electric currents enter the cathode section (negative), and electrons present in the electrolyte solution are transferred with the current to the anode section (positive), passing through the membrane. Eqs 4, 5 show the anode and cathode reactions. The hydrogen gives up its electrons to form positive ions (H^+^) and travel in the opposite direction and hydrogen is released as bubbles from the solution (Shiva Kumar and Himabindu, 2019).

$$Anode:2H_{2}O\to 4H^{+}+O_{2}+4e^{-}$$ (4)

$$Cathode\;:4H^{+}+4e^{-}\;\to 2H_{2}$$ (5)

The sustainability of the electrolysis process depends on the electricity supply where renewable energy sources such as solar, wind, or biomass are used to produce green hydrogen. Hence it emits oxygen as a byproduct that results in a zero-emission establishment that will have a positive contribution to the decarbonization of the wastewater treatment industry. Furthermore, an advanced treatment such as a reverse osmosis unit must be added to the wastewater treatment facility to ensure that the quality of treated effluent is suitable to be used in the electrolysis. However, many issues regarding the storage and transportation of the released hydrogen have been raised (Shiva Kumar & Himabindu, 2019).

## 5 Materials and methods of the study

### 5.1 Feasibility study based on Al ansab sewage treatment plant

The feasibility study was conducted at the Al Ansab STP which is managed by OWWSC. It is located in the governance of Muscat in Wilayat Bousher. The plant was commissioned in 1990 and was operated by Haya Water Company and in 2020, the STP management changed it to be under OWWSC.

Previously, the facility used the Conventional Activated Sludges (CAS) technology to treat the wastewater with a capacity of 20,000 m^3^/day. It included a tanker discharge area, physical pre-treatment facilities, secondary biological treatment, filtration, and chlorination. However, there were some drawbacks regarding the quality of treated effluent, the capacity of the plant, and the intensive chemical operations that might pose risks to human health and the environment. Consequently, a new membrane bioreactor (MBR) was installed to produce a higher treated effluent quality, operate more efficiently, and have a lower carbon footprint. It increased the capacity of the plant to 55,000 m^3^/day. Nevertheless, it is an energy-intensive process due to the additional water pumping system, and there is a risk of membrane fouling that can result in increased costs of maintenance and operations (Al-Wahaibi et al., 2021).

**FIGURE 4:**
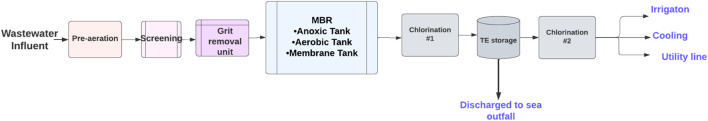
Al Ansab sewage treatment plant model.

An MBR system consists of four main areas: the head works area, biological treatment process area, treated effluent area, and sludge dewatering unit. The sources of wastewater are only domestic sources from tankers or pumping. First, the wastewater is collected in the headworks area where the primary treatment occurs. The pre-aeration units for the raw sewage keep the solid contaminants in suspension by using air diffusers and screening removes solid particles greater than 3 mm, while the grit removal unit removes fats and grease. Also, this area includes an odor control unit process to trap the noxious gases of hydrogen sulfide and ammonia, and a chemical storage and dosing process unit for caustic soda (sodium hypochlorite). In the biological treatment area, the biological membrane filtration process is used to reduce biological pollutants. To manage the produced waste, a sludge storage and dewatering system were employed by placing the sludge and taking it by a belt for thickening and dewatering. The sludge is utilized to produce compost under the brand name, KALA. The treated effluent area includes disinfection by dosing with chlorine to eliminate any organics that affect human health before and after storing the TE. Figure 4 shows the wastewater treatment in the Al Ansab STP used in this study. The plant receives 95,000 m^3^/day of wastewater and produces 92,800 m^3^/day of TE. Of the treated effluent produced, 82,200 m^3^/day is currently utilized for cooling and irrigation purposes and the other 9,800 m^3^/day is discharged into the sea. (Source: OWWSC).

**FIGURE 5:**
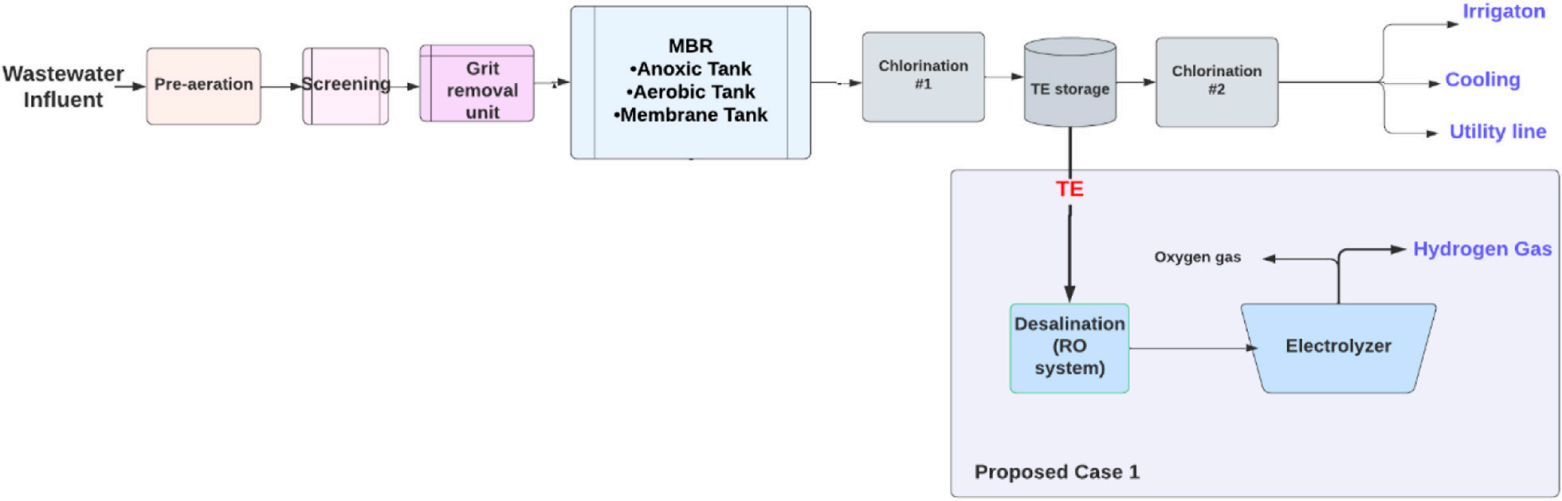
The Al Ansab sewage treatment plant with water reclamation opportunities.

In light of the above, there is an option for reusing TE that could be proposed for the Al Ansab STP, in order to utilize the 9,800 m3/day of water that is discharged to the sea daily. Figure 5 demonstrates the proposed cases for resource recovery. Case 1 shows green hydrogen production using electrolysis.

#### 5.1.1 Market analysis for green hydrogen production

**FIGURE 6:**
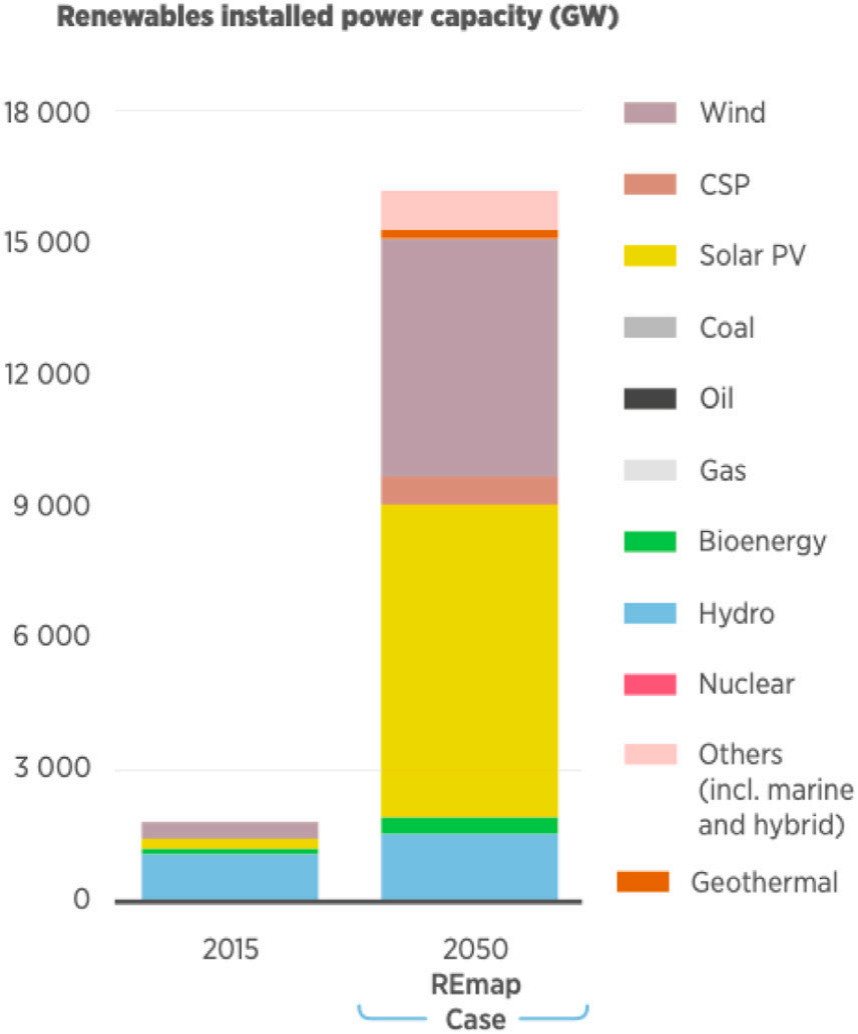
Energy resources installed in 2015 *versus* 2050 (Taibi et al., 2018).

The world has shifted its intentions in energy generation from using non-renewable energy sources such as fossil fuels to introducing greener options by utilizing renewable energy from solar and wind. Figure 6 illustrates the renewable energy resources that are planned to be installed by the year 2050 in comparison to the year 2015 (Taibi et al., 2018).

Hydrogen gas has the potential to be a clean future energy source in the energy market. Recently, the need for decarbonization in many sectors has increased. Hydrogen can be considered one of the solutions as it can be used in many sectors such as transportation and chemical refineries that face many difficulties in reducing their emissions. Also, one of the notable features of producing hydrogen gas is that it can be blended with natural gas and inserted into the natural gas grid, contributing to decarbonizing gas networks globally (Taibi et al., 2018). Moreover, the demand for hydrogen is not limited to the energy sector. The chemical sector uses hydrogen in the production of ammonia and has a small demand for hydrogen for manufacturing steel, iron, glass, and electronic parts. In addition, the hydrogen-to-power concept is achieved through fuel cells which is a promising solution for maintaining an uninterrupted power supply or backup that can be used in different systems (Taibi et al., 2018).

In addition, current technological breakthroughs were able to produce, store and utilize hydrogen for different purposes. The conventional way to supply hydrogen was through fossil fuels like coal and natural gas. Yet, renewables have a great role in the production of green hydrogen, and they can be one of the options to store energy produced by renewables. Also, from a social aspect, people are becoming more conscious of environmental issues arising globally. Governments have shown positive support by showing a willingness to welcome the new hydrogen economy (IEA, 2019).

Though, there are some barriers that face the hydrogen industry. Current hydrogen production is supplied by fossil fuels industries and is a major producer of CO_2_ emissions. Also, another major barrier is that any low-carbon hydrogen production processes are considered costly. However, a reduction of 30% in the cost of using hydrogen production for renewable energy by the year 2030 is possible as reported by the International Energy Agency. Mass manufacturing of hydrogen production components could have a positive impact on cost reduction (IEA, 2019). Table 2 summarizes the previous points in a Strength Weakness Opportunities and Threats (SWOT) analysis matrix for hydrogen as a product. It is conducted to identify the strengths, weaknesses, opportunities, and threats of any outcome (Ren et al., 2017).

**TABLE 2:** TE loading rate and concentration of the Al Ansab STP (Source: OWWSC).

Parameter	Loading (kg/day)		Concentration (mg/L)
	Average annual	Peak (Max months)	
BOD	480	625	5
TSS	480	625	5
Total N	960	1,000	8
ORG-N	0	0	0
NH3-N	69	125	1
NO3-N	768	750	6
Total Alkalinity	—	—	200

### 5.2 Technical feasibility for green hydrogen production

Technical analysis is conducted in this study to determine if the proposed ideas for water reclamation are technically feasible to be implemented or not. It includes aspects of types of technologies and resources that were implemented to produce hydrogen from products and by-products generated from the Al Ansab STP.

#### 5.2.1 Electrolysis technology

**FIGURE 7:**
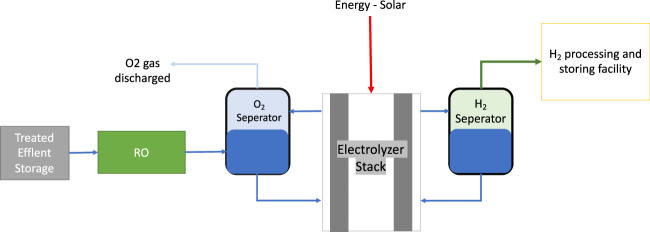
The model for electrolysis system flow process.

To implement electrolysis technology for producing green hydrogen, the system-level design and balance have to be followed. It can be divided into four main parts: the feed water, the electrolyzer stack, the hydrogen processing and storing facility, the oxygen gas separator, and a power supply system (IRENA, 2020). Figure 7 shows the typical system of design for hydrogen production from a purified water intake. Table 3 represents the loading and concentration of the TE as data provided by the company for Al Ansab STP, taking TE as a source of water that gets transported to the electrolysis system.

**TABLE 3:** SWOT analysis for hydrogen production.

**Strengths**	**Weaknesses**
Clean energy carrier	High costs
Decarbonization opportunities	Less commercialized technologies
Diversifying the economy	Storage and transportation challenges
**Opportunities**	**Threats**
High social acceptance	Lack of investment
Government support	Competition with non-renewable energy sources to produce hydrogen

The TE is considered a reliable source of water to supply the electrolysis for hydrogen production. According to a study that evaluates the sustainability of different water sources to supply the electrolysis process in Portugal using the sustainable value methodology (SVM), treated urban wastewater is considered reliable for short-term availability despite weather conditions and drought, and reliable for long-term availability against the future impacts of climate change. Also, it is freely accessible for reuse with paying minimum charges (Simões et al., 2021).

The final TE produced in the Al Ansab STP does not meet the standard to be used in an electrolyzer as it contains percentages of BOD that refers to the organic load in the water and because it goes through chlorination before storing. Highly purified input water is necessary for the efficiency of the electrolysis process. The more purified it is, the higher efficiency of hydrogen production. The addition of any dissolved components lowers the quality of water and may result in damaging the electrolysis membranes by scaling or fouling, leading to a shorter lifetime and faster degradation. It is stated that every 1 kg of hydrogen produced requires 9 kg of water to be supplied. The presence of inefficiencies in the electrolysis process will increase the demand for water required to 18–24 kg (IRENA, 2020).

According to the American Society for Testing and Materials (ASTM), water quality differs depending on the electrolyzer manufacturers. It identified the suitable electrolyzer input to be deionized water Type I or II or less purified with a conductivity of <5 μS/cm. Also, a demineralization step is included as a part of most electrolyzer equipment available in the market (Simões et al., 2021). Therefore, installing desalination technology such as RO or an ultrafiltration (UF) unit is needed to purify the water by using membrane technology with a high-pressure flow. The treatment level is identified depending on the electrolyzer type and technical specifications. RO technology includes partially permeable membranes that remove unwanted molecules and ions from water by applying higher pressure to overcome the osmotic pressure. It requires a high energy demand of 3–6 kWh/m^3^ and a high-pressure pumping system. However, UF has a more compact membrane design that is cost-effective, demands less maintenance, has an energy demand of only 0.025–0.1 kWh/m^3^, and produces less waste, 7% of the water feed, which results in less operational costs (Simões et al., 2021). Although UF is cost-effective and requires less energy, an RO system is considered the best option that produces water quality suitable for industrial applications and eliminates any scale formation, fouling, and corrosion to the equipment, especially in systems that work at high pressures like electrolyzers (Kehrein et al., 2021). However, commercially utilized electrolyzers such as Siemens PEM Silyzer electrolyzer units include a built-in water purification system (Siemens, 2020).

**FIGURE 8:**
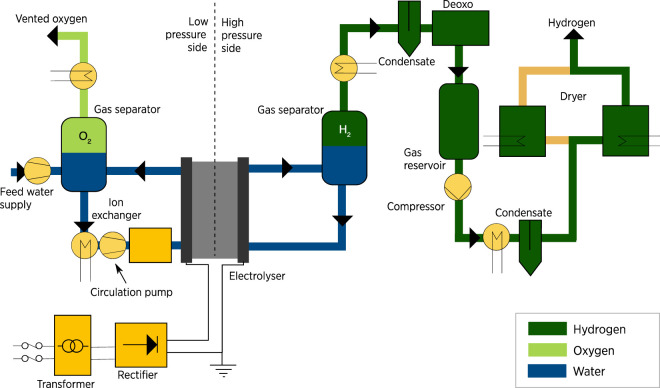
PEM electrolyser system. Source (IRENA, 2020).

The stack system differs depending on the type of electrolysis used. The PEM was selected as it is the newest commercialized technology with better features than alkaline electrolysis. Although the PEM stack is more complex than the typical design, it is considered a simpler design with less equipment than an alkaline electrolyzer system. Also, it is a more flexible system that can be applied to wider operating ranges with a shorter response time. PEM operates at a higher pressure of approximately 30 bar which requires additional unit operations on both the anode and cathode sides. Heat exchangers, circulation pumps, and pressure control and monitoring units are on the anode side. Also, there is a hydrogen processing unit on the cathode side that includes a gas separator. De-oxygenation is implemented to remove any excess oxygen in the hydrogen output, gas dryer, and compressor. The system design of PEM with the equipment is shown in Figure 8 (Taibi et al., 2018). The two products are oxygen, which is vented outside the system, and hydrogen, which gets processed and stored.

**FIGURE 9:**
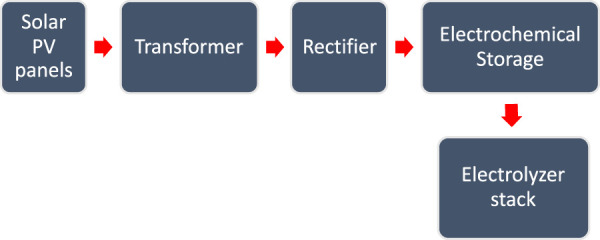
Power supply system for electrolyzer stack.

Thirdly, the power system that is used to supply electricity to the electrolyzer must be generated from renewable energy resources, in order to make it a green hydrogen production system. It demands the highest cost of all electrolyzer systems. In the case of the Al Ansab STP, the best option is solar energy, which was utilized as a source of electricity due to the location of Oman getting sunlight daily during the daytime. Solar panels are connected to a transformer which is connected to a rectifier for converting AC to DC. Electrochemical storage (battery) is installed to store the generated power and supply it to the electrolyzer as presented in the schematic flow in Figure 9. The optimum design of the power system depends on the efficiency of the electrolyzer and the flexibility of the system in handling the fluctuations of the solar energy, in case of the absence of direct sunlight due to shadowing, clouds, or nighttime (Taibi et al., 2018).

A potential economic study conducted to analyze solar to hydrogen production using PEM electrolysis was done for a different Wilayat in Oman. As the Al Ansab STP is located in Muscat, the solar PV energy result is 2,939 MWh/year which yielded 55,527 kg H_2_/year (Ahshan & Perea-Moreno, 2021). This can confirm the possibility of using the PV system with PEM technology to produce hydrogen in the Al Ansab STP. However, land use is one of the drawbacks of utilizing this technology as solar panels have to be placed in wider areas and the electrolyzer land area is based on the capacity of the plant. The area of the PEM electrolyzer can be reduced with a double-floor building design or smaller container units that hold multiple stacks of electrolyzers. A study conducted by the Institute for Sustainable Process Technology (ISPT) concluded that one GW PEM plant requires a maximum area of 0.08 km^2^ (8ha) (IRENA, 2020; ISPT, 2020).

### 5.3 Economic feasibility of the study

The economic analysis is considered the most important part of the feasibility study. The determination of the cash flows by conducting a cost-benefit analysis (CBA) and using the net present value (NPV) method is shown in Eq. 6 (Kehrein et al., 2020). From Eq. 6, r is the rate of interest or the discount rate, t is the time and n is the number of periods (number of years for the study). The lifetime n estimated for this water reclamation process is 10 years with a 5% discount rate (Kehrein et al., 2021). As shown in Eq. 7, NP is the net profit, and it is determined by subtracting the total benefits from the total costs of each year. Also, an inflation rate of 2.67% was selected according to what was published at the National Centre of Statistics and Information in Oman (NCSI, 2022).

$$NPV=\overset{n}{\underset{t=0}{\sum }}\frac{{NP}_{t}}{{\left(1+r\right)}^{t}}$$ (6)

$$NP=\sum B_{i}-\sum C_{i}$$ (7)

**FIGURE 10:**
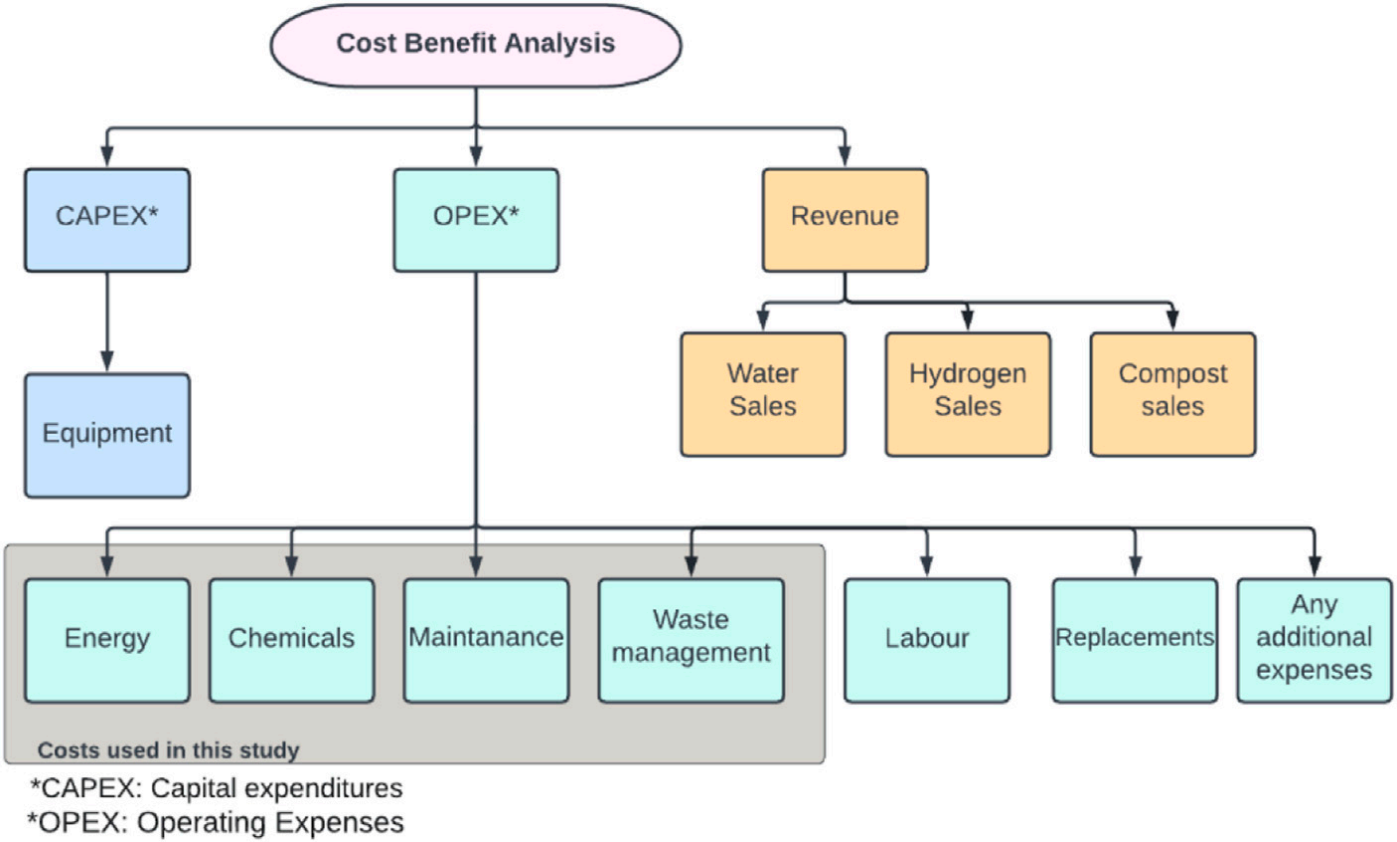
Cost-benefit analysis model.

A comparison is conducted between the conventional case of the Al Ansab STP and the two proposed technologies to produce hydrogen to determine their feasibility. The CBA elements of capital expenses (CAPEX), operating expenses (OPEX), and revenue are identified in Figure 10. CAPEX is defined as the capital expenditures of the equipment costs. OPEX is the operational costs, and they might vary with time. These costs include energy, chemicals, maintenance, replacement, labor, waste management, and any additional costs. The costs used for the conventional case of the Al Ansab STP were provided by the plant operators that work under OWWSC. The net savings is calculated between the revenue generated from the conventional and the proposed cases. Similar CBA studies were done for hydrogen production using different technologies of AWE, PEM, and SOE as summarized in Table 4.

**TABLE 4:** Different green hydrogen technologies with cost and conversion efficiency.

Year	Technology	CAPEX costs, conversion efficiency	References
2015	AWE	1,150 $/KW, 70%	Glenk and Reichelstein, (2019)
	PEM	1,322 $/kW, 60%	Glenk and Reichelstein, (2019)
	SOE	8,571 $/KW, 80%	Winkler-Goldstein and Rastetter, (2013)
2025	AWE	1,065 $/KW, 70%	Glenk and Reichelstein, (2019)
	PEM	1,065$/KW, 60%	Glenk and Reichelstein, (2019)
	SOE	1,057 $/kw, 80%	
2030	AWE	737 $/KW, 70%	Jülch, (2016)
	PEM	737 $/KW, 60%	
	SOE	737$/KW, 80%	

## 6 Results and discussion

### 6.1 Technical results

**FIGURE 11:**
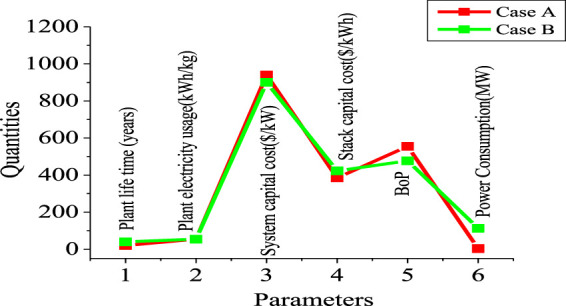
Technical parameters of Cases A and B.

Table 5; Figure 11 show the technical data that were obtained and used during this study. Two electrolysis systems were used: Case A with a lower capacity of 1,500 kg of H_2_ per day and Case B with a higher capacity of 50,000 kg of H_2_ per day. This helped demonstrate the robustness of the system by comparing two capacities as usually used in an H_2_A study (hydrogen using electrolyzer) with technical parameters.

**TABLE 5:** Technical data for the electrolyzers.

PEM H_2_ case technical parameters	A	B
Plant capacity (kg H_2_/day)	1,500	50,000
Plant lifetime (years)	20	40
Plant electricity usage (kWh/kg)	54.6	54.6
System capital cost ($/kW)	940	900
Stack capital cost ($/kWh)	385.00	423
BoP	555	477
Power consumption (MW)	3.4	113.1

### 6.2 Economic results

Cash flow tables were generated using Microsoft Excel to validate the CBA and NPV calculations. The usual revenue expected from a wastewater treatment facility is from treated water sales and compost sales. OWWSC are selling the TE with a price of 0.220 RO/m^3^ and they produce compost in the brand of KALA and annual revenue reaches 500,000 OMR/year. Table 6 indicates the costs and revenues provided by OWWSC for the Al Ansab STP.

**TABLE 6:** Prices from the Al ansab STP. Source: (OWWSC).

Conventional method (irrigation, cooling, and utility) (OMR/year)	
CAPEX	
Equipment (initial investment)	80,000,000
OPEX	
Labor	—
Energy	1,046,910.67
Chemicals	246,233.54
Waste management	70,000
Maintenance	30,000
Total OPEX	1,563,505.21
Benefits	
Compost	500,000
Reclaimed water prices	6,520,360
Total Benefit (total profit)	7,020,360
**NPV for 10 years study period (million OMR)**	**50.23**

Green hydrogen production costs vary depending on the technology used. The proposed technologies were a PEM electrolysis system which provided additional revenue from selling the produced hydrogen gas. Currently, the prices of hydrogen in Oman could not be determined, therefore the prices of hydrogen from the Department of Energy in the United States United States published reports based on the cost of hydrogen produced from a PEM electrolyzer using solar energy (PV) as a source of energy were used. They were 6$/kg of H_2_ which is equal to 2.31 OMR/kg of H_2_ (Vickers et al., 2020). The CAPEX, OPEX, revenue, and NPV results are shown in Table 7, which were calculated and collected from different articles (James et al., 2013; Tapia-Venegas et al., 2015; Navas Carlos, 2017; Saba et al., 2018; IRENA, 2020; Kehrein et al., 2021).

**TABLE 7:** CBA and NPV results.

Annual values (OMR)		Al ansab STP conventional case	Al ansab STP + PEM electrolysis system (Case A)	Al ansab STP + PEM electrolysis system (Case B)
Total CAPEX		80,000,000.00	1,246,440.00	39,698,100.00
Total OPEX		1,393,144.21	1,418,073.01	2,187,106.21
Total Revenue	Year 1	7,020,360.00	8,301,510.00	49,725,360.00
	Year 5	37,123,724.98	53,944,335.89	600,221,357.76
	Year 10	79,886,223.90	224,570,287.92	4,919,138,444.66
NPV for 10 years study period (Million OMR)		**50.23**	147.63	3,308.44

For PEM, two possible options were studied, one with a lower capacity of 1,500 kg H_2_/day (Case A) and a higher capacity of 50,000 kg H_2_/day (Case B). The water consumption in the systems was 27 m^3^/day and 900 m^3^/day, respectively (James et al., 2013). Combined with the Al Ansab STP conventional case, hydrogen sales increased the revenue to 8.30 million OMR/year, and 49.73 million OMR/year, respectively.

**FIGURE 12:**
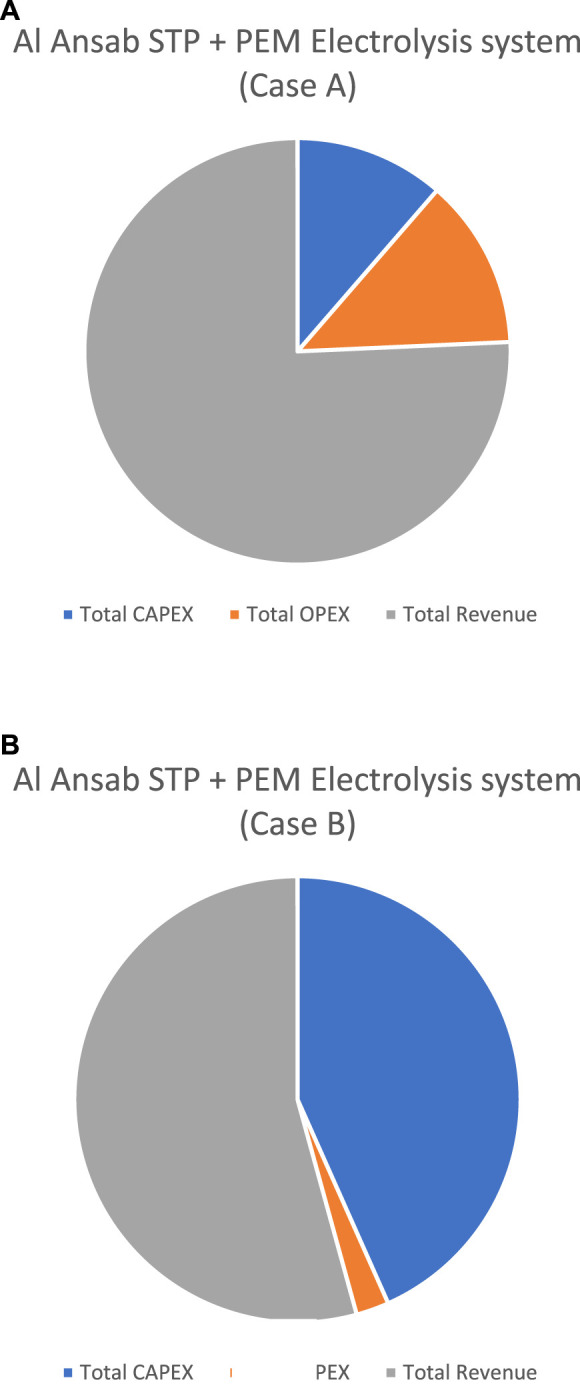
Economical results of the Al Ansab case study. **(A)** Al Ansab STP + PEM Electrolysis system (Case A). **(B)** Al Ansab STP + PEM Electrolysis system (Case B).

Moreover, NPV analysis was conducted for all the cases as shown in Table 7; Figure 12, for a 10-year study period. A positive NPV resulted from all the cases which indicates that the calculated earned benefits from the sale of treated water, compost, and hydrogen compensated for the investment costs. The highest NPV is from Case B for the PEM electrolyzer system because of the large plant capacity of 50,000 kg H_2_/day. However, a positive NPV can also mean higher CAPEX and OPEX costs to achieve a valid revenue as the PEM stacks’ capital costs are relatively high and demand a greater energy cost. Hence, hydrogen can be achieved with lower-cost processes like in Case A.

Although the water consumption is less than the reclaimed water from the Al Ansab STP (9,800 m^3^/day), a valid amount of revenue was gained from Case A and B. However, an assumption can be made that if 9,800 m^3^ of water/day entered the electrolyzer system, it will result in the generation of approximately 544,444 kg of H_2_/day and an annual revenue gain of roughly 460 million OMR/year.

Moreover, the Al Ansab STP utilizes 81,200 m^3^/day of TE in irrigation and cooling, and sludge is sold as compost for both, generating a combined revenue of 7.02 million OMR/year. Investigating the same quantity of TE to be used instead for hydrogen production by electrolysis, the revenue of 3.8 billion OMR/year will be gained which is much greater than the conventional case. On the other hand, if the 9,800m^3^/day of water discharged into the sea was used for irrigation and cooling instead, alongside selling compost, a smaller revenue of 1.29 million OMR/year would be obtained.

## 7 Conclusion and the future directions

The need for water reuse is imperative due to global environmental challenges that cause impediments to the availability of sustainable water for all. Consequently, the introduction of more feasible water reclamation technology is of high importance because large amounts of high-quality reusable water could be recovered and used for many applications.

Green hydrogen has various utilizations in the future of energy as it is well known for its sustainable energy system. It is produced from processes that utilize water and biomass which will open the horizon for future resource recovery opportunities from different sectors. Although clean hydrogen production processes are still new and developing, the increase in hydrogen demand and the flourishing of the market will make it easier to shift to large-scale production.

This paper demonstrated the technical and economic feasibility of green hydrogen production in Oman using treated effluent from wastewater. The Al Ansab sewage treatment plant in Bousher was used for the study. The technical aspect of the study was evaluated using two electrolysis systems, while the economic feasibility was carried out by determining the cash flows for a cost-benefit analysis using the net present value approach. The results show that the water consumption is less than the reclaimed water from the Al Ansab sewage treatment plant of 9,800 m^3^/day, using two cases with different capacities. Furthermore, approximately 544,444 kg of H_2_/day would be generated, creating room for an annual revenue gain of approximately 460 million OMR/year. Although capital costs of green hydrogen production are considered high, the revenue gained from selling hydrogen will cover the initial investment costs in a short period.

In conclusion, Oman, alongside many countries, is encouraging green hydrogen production. The treated effluent produced should be considered a valuable source of water for many applications, especially for industrial uses as they require large water demand, instead of discharging it into the sea, a huge waste of resources. Moreover, the wastewater management strategy employed in this paper would contribute to the global goal of fighting climate change and achieving decarbonization in many sectors, encouraging the world to reach net zero emissions.

## Data Availability

The original contributions presented in the study are included in the article/Supplementary Material, further inquiries can be directed to the corresponding authors.
